# First Shotgun Metagenomic Characterization of the Gut Microbiome of the Endangered Longhorn Beetle *Callipogon relictus* (Coleoptera: Cerambycidae)

**DOI:** 10.3390/insects17101016

**Published:** 2026-10-01

**Authors:** Alexander Kuprin, Vladislava Baklanova, Anastasia Rechits

**Affiliations:** 1 Federal Scientific Center of the East Asia Terrestrial Biodiversity, Far Eastern Branch of the Russian Academy of Sciences, Vladivostok 690022, Russia; veremenko.vs09@gmail.com; 2 Institute of the World Oceans (School), Far Eastern Federal University, Russky Island, Vladivostok 690090, Russia; rechitc.as@dvfu.ru

**Keywords:** beetle gut microbiome, Cerambycidae, *Callipogon relictus*, developmental stage, shotgun metagenomics, wood-feeding beetles, symbiotic associations

## Abstract

The gut microbiome of *Callipogon relictus*, a rare member of the family Cerambycidae, was characterized for the first time using shotgun metagenomic sequencing. The aim of this study was to compare the taxonomic diversity and structure of the gut microbial communities in larvae and adults. Bacteria dominated the metagenomic profiles, accounting for 92.47% of the taxonomic composition in adults and 85.31% in larvae. Proteobacteria predominated in the bacterial component of both pooled profiles, accounting for 87.88% and 89.43%, respectively. The pooled adult profile had the highest relative abundances of Gammaproteobacteria and Enterobacterales, whereas the pooled larval profile had higher relative abundances of Alphaproteobacteria and Rickettsiales. In the pooled samples analyzed, 155 bacterial and 69 fungal genera were detected in the larval profile, compared with 85 and 17 genera, respectively, in the adult profile; the fungal component accounted for 10.46% of the larval profile and 0.18% of the adult profile. The two profiles shared 77 bacterial, 17 fungal, and 15 protist genera. The virus-like/retroelement sequence fraction was dominated by sequences related to *Errantivirus*, a member of the family *Metaviridae*. Because *Metaviridae* comprise LTR retrotransposons, this signal was interpreted as endogenous retroelement DNA rather than evidence of a free-virus gut virome. These results provide a preliminary description of a shared bacterial core accompanied by differences between the pooled profiles in lower-rank bacterial composition and in the structure of the fungal and protist components of the *C. relictus* microbiome.

## Simple Summary

In saproxylic insects, the transition from the larval stage to adulthood is accompanied by changes in habitat and diet that may affect the gut microbiome. We characterized these developmental differences in the rare relict longhorn beetle *Callipogon relictus* using shotgun metagenomic sequencing. A comparison of gut samples from larvae inhabiting decaying Japanese elm wood and from adults showed that Proteobacteria was the predominant bacterial phylum in both pooled samples. However, the taxonomic composition of the microbial communities differed at the class, order, and genus levels. In the pooled adult sample, Gammaproteobacteria and Enterobacterales had the highest relative abundances, whereas in the pooled larval sample showed higher relative abundances of Alphaproteobacteria and Rickettsiales. The pooled larval sample contained nearly twice as many bacterial genera and more than four times as many fungal genera as the pooled adult sample; fungi also accounted for a larger proportion of the larval profile. The protist component differed not in the number of genera but in the dominance pattern: trypanosomatid-related sequences predominated in adults, whereas gregarine-related sequences predominated in larvae. Thus, despite pronounced differences between the pooled profiles in bacterial taxonomic composition and in the structure of the fungal and protist components, both profiles contained a shared bacterial core of the *C. relictus* gut microbiome.

## 1. Introduction

The insect gut microbiome is an important factor influencing the host’s ability to exploit nutritionally challenging resources. This role is particularly important for xylophagous insects, because wood is a mechanically robust, nitrogen-poor substrate in which carbohydrates are embedded within a matrix of cellulose, hemicellulose, and lignin [1]. Longhorn beetles (Coleoptera: Cerambycidae) occupy a wide range of wood-associated microhabitats. Larvae develop under the bark, in sapwood, heartwood, roots, or in dead and weakened wood, whereas adults of different taxa may feeding habits that differ from those of larvae, consuming sap, bark, or pollen, or may be non-feeding [2,3,4].

Recent studies indicate that plant cell-wall degradation in cerambycids depends not only on endogenous insect enzymes but also on bacterial and fungal enzymatic activity, as well as on microbial pathways involved in nitrogen fixation, vitamin and amino-acid biosynthesis, sterol production, and detoxification of plant secondary metabolites [2,5,6,7]. Metagenomic and metatranscriptomic analyses of longhorn beetle larvae have identified microbial genes potentially involved in lignin and hemicellulose degradation, xylose fermentation, and the provision of essential nutrients lacking in a wood-based diet [8,9]. These findings are relevant not only to understanding the nutritional physiology of xylophagous beetles but also to investigating the evolutionary transition of insects to nitrogen-poor, lignocellulose-rich substrates.

The relict longhorn beetle *Callipogon relictus* Semenov, 1899 is the only representative of the genus *Callipogon* in the Palearctic and is a rare, protected species of Northeast Asia [10,11]. Available data indicate that the development of *C. relictus* is closely associated with decaying wood of broad-leaved trees. Its natural life cycle may span several years, and larvae use the wood substrate as their primary habitat and food source [12]. In this context, the gut microbiome may represent an additional, previously unexplored component of adaptation in longhorn beetles, reflecting both host phylogenetic history and local conditions of larval development, including tree species, wood-decay stage, and fungal colonization. Therefore, comparison of the gut microbiomes of larval and adult *C. relictus* may help determine which microbial components are retained during metamorphosis, acquired from the environment, or associated with the physiological requirements of a particular developmental stage [7]. Metagenomic analysis of *C. relictus* may provide insight into feeding and development on nitrogen-poor substrates, identify potential microbial markers of developmental stage, and inform the evaluation of artificial media and ex situ rearing conditions relevant to conservation and population-restoration programs. From a practical perspective, data on the capacity of gut bacteria from saproxylic insects to transform lignin may inform future approaches to the bioproduction of value-added chemicals from lignin-rich waste [13].

The aim of this study was to characterize the taxonomic diversity of the gut microbiome in larval and adult *C. relictus* and to describe taxa detected in association with each developmental stage. We hypothesized that the larval gut microbiome would contain a greater number of bacterial and fungal taxa potentially associated with wood degradation, nitrogen fixation, and essential-nutrient biosynthesis, whereas the adult profile would reflect the shorter adult activity period, dietary differences, and post-metamorphic restructuring of the gut. We further hypothesized that taxa shared between the pooled larval and adult profiles may represent a conserved component of the microbiome; however, their symbiotic functions require independent validation.

## 2. Materials and Methods

### 2.1. Sample Collection and Preparation

Larvae of *Callipogon relictus* were collected in May 2026 from decaying wood of a single Japanese elm (*Ulmus japonica*) in a valley elm–broadleaved forest within the Ussuriysk Urban District (Primorsky Krai, Russia) [14,15]. Adults were collected at the same locality from large-stemmed elms in July 2026.

For each developmental stage, three individuals were sampled and their guts were pooled: sample S15703Nr1 (adults: two males and one female) and sample S15703Nr2 (larvae: three individuals). Sampling of this red-listed species was conducted in accordance with permit No. R027-00113-77/04609055 issued on 19 March 2026. All larvae were identified as sixth-instar individuals based on head capsule width [14]. Three individuals per stage were pooled because a single specimen did not yield sufficient microbial DNA for shotgun metagenomic sequencing, and increasing the sample size would have required additional destructive sampling, which is not permitted for this protected species. Pooling was therefore necessary but precluded assessment of individual variation and sex-specific effects. The adult sex ratio (two males and one female) reflects opportunistic collection during the short flight period.

Samples were transported to the laboratory in sterile containers under refrigeration (4 °C) within 6 h of collection. Adults were not fed and were maintained at room temperature (22 ± 2 °C) for no more than 24 h prior to euthanasia to minimize diet-related changes in the microbiome. Larvae were kept in their native wood at 6 °C until dissection (no more than 48 h).

Each specimen was rinsed three times for 30 s with sterile filtered water to remove external contaminants and then immersed in 70% ethanol for 2 s for surface disinfection. Samples were subsequently placed on a sterile, chilled surface, and the abdominal cavity was opened using sterile dissection instruments (scalpel, forceps, scissors) pretreated with 70% ethanol. The gut (from the pyloric appendages to the hindgut, including the midgut and hindgut) was carefully excised and separated from surrounding tissues and hemolymph using sterile forceps. Gut contents were not removed; the entire gut was analyzed as a single unit. Excised guts were placed in 96% ethanol and stored at −20 °C until DNA extraction (no more than 2 weeks).

Total DNA was extracted using the E.Z.N.A.® Mollusc DNA Kit (Omega Bio-tek, Norcross, GA, USA), which was selected for its efficient lysis of chitinous and polysaccharide cell walls of fungi and bacteria. Samples were homogenized in lysis buffer with proteinase K (55 °C, 3 h) using sterile pestles. A negative extraction control (reagents without sample) was processed through all extraction and subsequent sequencing steps, and no contamination was detected.

### 2.2. Quality Control and Library Preparation

The quality of the extracted DNA was assessed fluorometrically using the Quant-iT™ PicoGreen™ dsDNA Assay Kit (Thermo Fisher Scientific, Waltham, MA, USA) on a Qubit™ 4 Fluorometer (Thermo Fisher Scientific) according to the manufacturer’s instructions. DNA concentrations were 42.1 ng µL^−1^ for sample S15703Nr1 and 36.5 ng µL^−1^ for sample S15703Nr2, corresponding to total DNA amounts of 0.716 µg and 0.621 µg, respectively. All samples passed quality control and were approved for further processing.

Libraries were prepared using the Illumina DNA Prep (M) Tagmentation Kit (Illumina, San Diego, CA, USA), which includes unique dual indices (UDI), according to the manufacturer’s protocol on a Bio-Rad T100 Thermal Cycler (Bio-Rad Laboratories, Hercules, CA, USA). A total of 100.00 ng of DNA was used for library preparation. Library quality and size distribution were assessed according to the manufacturer’s recommendations. Free adapters were removed during library preparation, libraries were stored individually at −20 °C, and pooling was performed immediately prior to sequencing.

Sequencing was performed on an Illumina NovaSeq X Plus platform (paired-end 2 × 150 bp) at CeGaT GmbH (Center for Genomics and Transcriptomics, Tübingen, Germany). Read quality was evaluated using FastQC (v0.11.5-cegat) [16]. The proportion of reads with Q30 quality was 93.88%, indicating high sequencing quality (error probability < 1/1000).

### 2.3. Bioinformatic Data Processing

Read demultiplexing was performed using Illumina bcl2fastq software (v2.20). For sample S15703Nr1, 56.568 million reads (5.655 Gb) were obtained, and for sample S15703Nr2, 69.216 million reads (6.926 Gb) were obtained. Adapters were removed, and low-quality sequences were filtered using Trimmomatic (v0.39) [17] with the following parameters: Phred score < 20 in a sliding window of 4 bp, and a minimum read length of 50 bp after trimming. All bioinformatic analyses were performed on a standard Linux server.

Taxonomic analyses were conducted as follows: 10 million adapter-filtered forward reads (a random subset was selected for standardization) were aligned against the filtered RefSeq protein database (version 94; vertebrate and plant sequences excluded) using DIAMOND (v2.0.15) in BLASTX mode (e-value = 1 × 10^−5^) [18]. Taxonomic assignment was performed using the Lowest Common Ancestor (LCA) algorithm implemented in MEGAN6 Ultimate Edition (v6.18.7) with the following parameters: min. support = 1, min. score = 50, top percent = 10 [19]. Only taxa with a relative abundance greater than 0.01% were retained to reduce false-positive assignments.

Reads corresponding to host DNA (Arthropoda) and vertebrate contaminants (Chordata) (see Table S1) were excluded from further analysis according to the LCA algorithm (assigned to taxa above the threshold). The remaining reads were used for taxonomic analysis.

Reads that could not be assigned to any of the taxonomic domains (Bacteria, Archaea, Eukaryota, Viruses) with sufficient LCA confidence were classified as “unidentified”. In the present dataset, such reads accounted for less than 0.01% of the total reads in both samples (Table 1). They were therefore excluded from diversity analyses but were included in calculations of the overall microbiome structure; owing to their very low proportion, their exclusion had a negligible effect on the results.

### 2.4. Analysis of Taxonomic Composition

For several taxonomic levels (phylum, class, order, family, and genus), stacked bar plots were constructed using the ggplot2 package (v3.3.5) in R (v4.0.4) [20]. Data were normalized to 100% for each sample separately. Taxa were arranged in descending order of total abundance (most abundant taxa at the bottom of the plot). Taxa with uncertain taxonomic assignments were verified and, where possible, updated according to the current NCBI classification as of 2026; where uncertainty persisted, the designation “–” was used. Taxa not classified to the corresponding level were grouped as “unclassified” in tables at higher taxonomic levels.

Venn diagrams illustrating the overlap of genera were constructed using the online platform InteractiVenn (https://interactivenn.net/ accessed on 10 August 2026) [21]. Complete lists of genera detected in each group, with a relative abundance threshold > 0.01%, were uploaded to the platform. Only unique genera were used to construct the Venn diagrams.

### 2.5. Alpha and Beta Diversity

To describe within-community diversity of the *C. relictus* microbiome, alpha diversity indices were calculated at the genus level for bacteria, fungi, protists, and virus-like/retroelement sequences (Table S6a–d) using the following metrics: richness (number of genera with non-zero read counts), Shannon index (accounting for richness and evenness of abundance distribution), Simpson index (probability that two randomly selected reads belong to different taxa), and Pielou’s evenness (degree of uniformity in the distribution of abundances among taxa).

To describe the degree of dissimilarity between the microbial communities of adult and larval *C. relictus*, beta diversity indices were calculated at the genus level for bacteria, fungi, protists, and virus-like/retroelement sequence (Table S6a–d) using two presence/absence indices, the Jaccard index and the Sørensen index, and one abundance-based index, the Bray–Curtis dissimilarity. The Jaccard and Sørensen indices reflect the proportion of unique taxa in each stage and range from 0 (complete similarity) to 1 (complete dissimilarity). The Bray–Curtis dissimilarity was calculated from absolute counts and also ranges from 0 (complete similarity) to 1 (complete dissimilarity).

Analyses were performed using the vegan package (v2.5-7) in R (v4.0.4) [22]. Because each stage was represented by a single pooled sample (n = 1 per stage), these indices are descriptive only, and no statistical significance was assessed.

### 2.6. Statistical Analysis

Owing to sample pooling (n = 1 per group) and the absence of biological replicates, no statistical tests for group comparisons were performed. Only descriptive results are presented: relative abundances of taxa, diversity indices, and beta-diversity distances. Differences between stages mentioned in the text should be regarded as observed patterns rather than statistically confirmed effects. The obtained data require validation in studies with larger numbers of biological replicates.

The detection threshold for taxa was set at a relative abundance ≥ 0.01% to reduce the influence of sequencing artifacts. Taxa with abundances below this threshold were excluded from the analysis.

## 3. Results

### 3.1. Overall Structure of the C. relictus Microbiome

Analysis of the distribution of reads across domains (Table 1) showed that bacteria constituted the core of the *C. relictus* gut microbiome in both pooled samples: the adult sample comprised two males and one female (92.47%), whereas the larval sample comprised three individuals (85.31%). The fungal component was markedly enriched in the pooled larval sample (10.46% vs. 0.18% in the pooled adult sample), representing an approximately 58-fold higher proportion than in adults. Protists (5.99% in adults, 2.53% in larvae) and virus-like/retroelement sequences (1.36% in adults, 1.70% in larvae) were present at lower proportions. Archaea were not detected. Unidentified sequences accounted for less than 0.01% in both samples.

### 3.2. Bacterial Component

**Figure 1:**
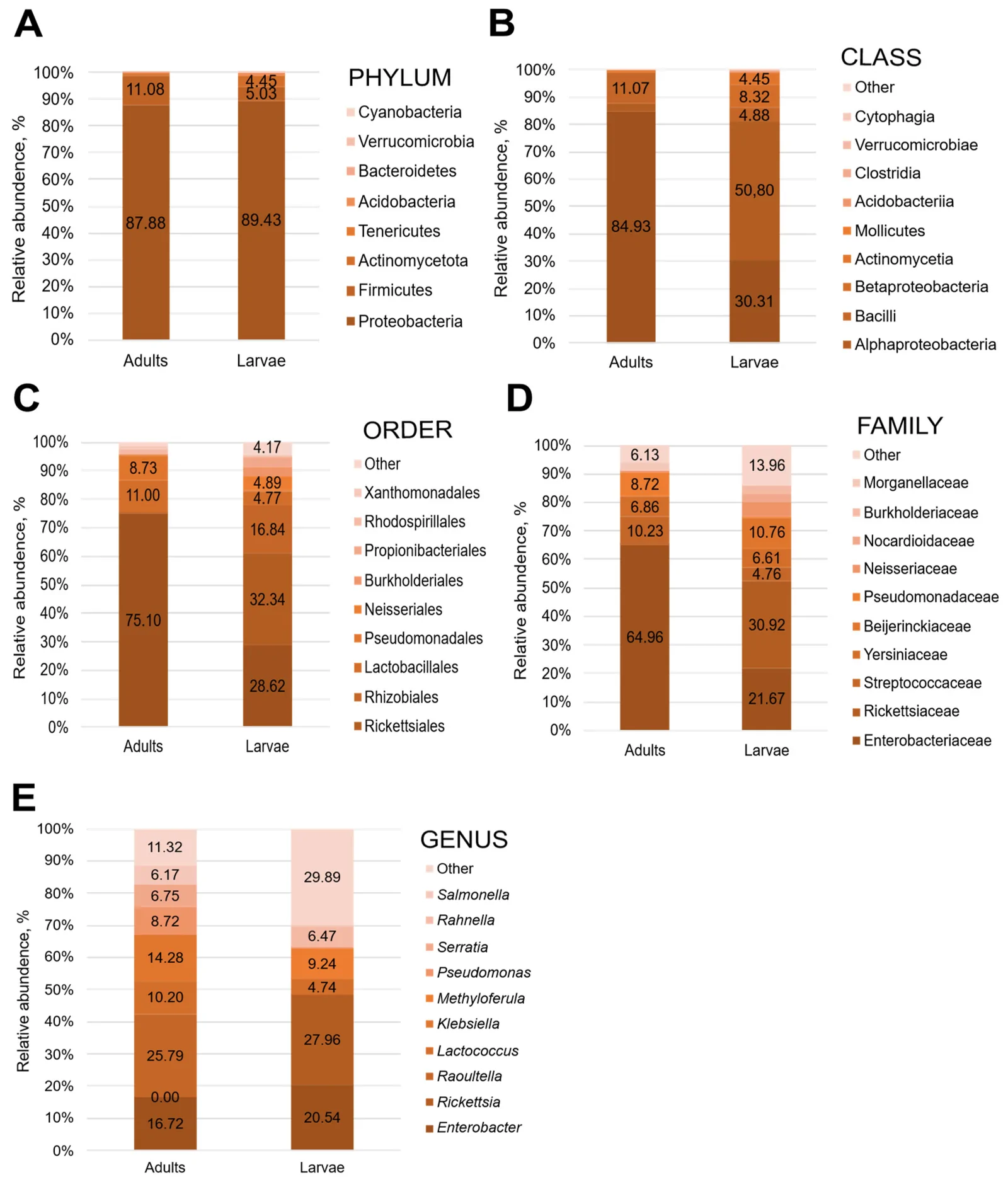
Taxonomic structure of the bacterial community in the gut of *Callipogon relictus* adults and larvae. Relative abundances (%) of bacterial taxa at the phylum (**A**), class (**B**), order (**C**), family (**D**), and genus (**E**) levels are shown for pooled samples (adults: two males and one female; larvae: three individuals; n = 1 per stage). Samples were collected from Japanese elm (*Ulmus japonica*) in a valley elm–broadleaved forest, Ussuriysk Urban District, Primorsky Krai, Russia: larvae from decaying wood, adults from large-stemmed trees. The top 10 taxa are shown; remaining taxa are grouped as “Other”. Values are descriptive; no statistical comparison between stages was performed.

In the pooled samples, the taxonomic composition of the bacterial component differed between the adult and larval profiles of *C. relictus* (Figure 1). The total number of bacterial reads was 193,768 in the pooled adult sample and 52,301 in the pooled larval sample. The taxonomic structure of the bacterial community differed between the pooled profiles at all taxonomic levels.

At the phylum level (Table S2a), Proteobacteria was the dominant group in both pooled profiles (87.88–89.43%). The proportion of Firmicutes decreased from 11.08% in adults to 5.03% in larvae, whereas the share of Actinomycetota increased from 0.30% to 4.45%. Tenericutes were present only in adults (0.73%), whereas Acidobacteria, Bacteroidetes, Verrucomicrobia, and Cyanobacteria were represented predominantly or exclusively in the larval stage.

At the class level (Table S3a), the dominance of Gammaproteobacteria in adults (84.93%) was replaced in larvae by a decrease to 30.31% and a sharp increase in Alphaproteobacteria (from 2.75% to 50.80%), driven primarily by Rickettsiales and Rhizobiales, as well as Betaproteobacteria (from 0.20% to 8.32%). The class Bacilli decreased from 11.07% to 4.88%, whereas Actinomycetia increased from 0.30% to 4.45%, and Acidobacteriia appeared in larvae (0.68%).

At the order level (Table S4a), Enterobacterales, which dominated in adults (75.10%), decreased in larvae to 28.62%, giving way to Rickettsiales (from 0.14% to 32.34%), Rhizobiales (from 0.42% to 16.84%), and Burkholderiales (from 0.03% to 3.43%), as well as the emerging Neisseriales (4.89%). Lactobacillales decreased from 11.00% to 4.77%, whereas Acidobacteriales (0.68%), Verrucomicrobiales (0.15%), and Nostocales (0.03%) were present exclusively in larvae.

At the family level (Table S5a), Enterobacteriaceae decreased from 64.96% in adults to 21.67% in larvae, yielding dominance to Rickettsiaceae (from 0% to 30.92%) and the emerging Beijerinckiaceae (10.76%), whereas Pseudomonadaceae decreased from 8.72% to 0.49%. Nocardioidaceae (from 0.01% to 3.02%), Burkholderiaceae (from 0.02% to 2.92%), Rhizobiaceae (from 0.01% to 2.60%), and Bradyrhizobiaceae (from 0.03% to 2.23%) increased in larvae. Morganellaceae and Spiroplasmataceae were represented almost exclusively in adults, whereas Acidobacteriaceae, Sphingomonadaceae, and Verrucomicrobiaceae were found only in larvae. The proportion of other families increased from approximately 3% to approximately 12%, reflecting increased taxonomic diversity at the larval stage.

The largest differences in relative abundance between the pooled profiles were observed at the genus level (Table S6a). In the pooled adult sample, the genera with the highest relative abundances were *Raoultella* (25.79%), *Enterobacter* (16.72%), *Klebsiella* (14.28%), *Lactococcus* (10.20%), *Pseudomonas* (8.72%), *Serratia* (6.75%), and *Salmonella* (6.17%). In the pooled larval sample, the relative abundances of these genera were lower, and *Rickettsia* (27.96%), which was nearly absent in the pooled adult sample, showed the highest relative abundance. *Enterobacter* was the second most abundant genus in the pooled larval sample (20.54%). Genera such as *Methyloferula* (9.24%), *Rahnella* (6.47%), *Neisseria* (4.89%), *Orientia* (2.96%), *Nocardioides* (2.90%), *Rhizobium* (2.33%), *Bradyrhizobium* (1.29%), and other representatives of Alphaproteobacteria and Actinomycetota were detected predominantly or exclusively in the pooled larval sample. Genera such as *Morganella* (1.89%), *Spiroplasma* (0.73%), *Gibbsiella* (0.39%), and others were detected exclusively in adults. The total proportion of other genera was about 12% in the pooled larval sample compared with 2% in the pooled adult sample, consistent with the higher number of detected genera in larvae.

### 3.3. Fungal Component

**Figure 2:**
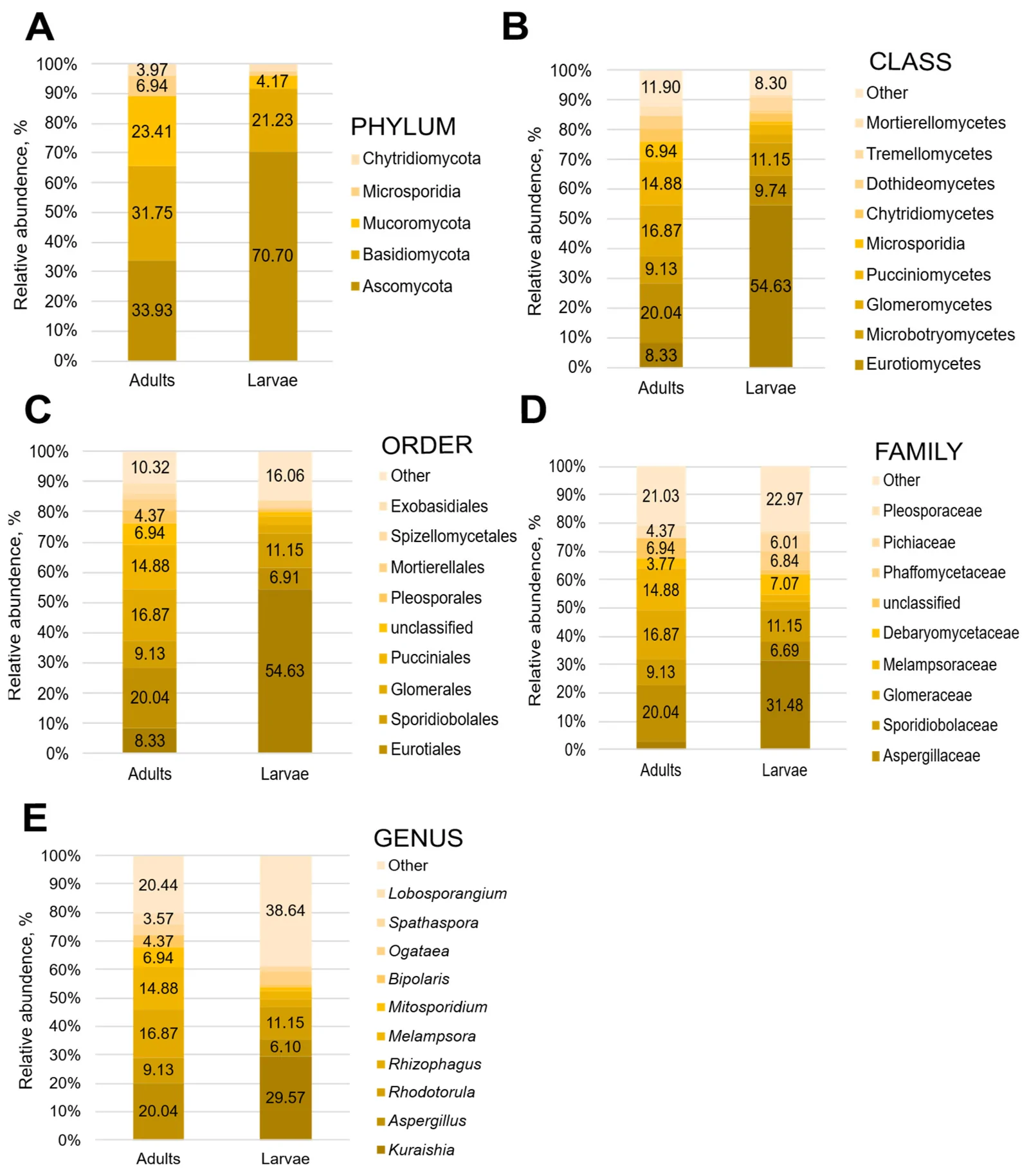
Taxonomic structure of the fungal community in the gut of *Callipogon relictus* adults and larvae. Relative abundances (%) of fungal taxa at the phylum (**A**), class (**B**), order (**C**), family (**D**), and genus (**E**) levels are shown for pooled samples (adults: two males and one female; larvae: three individuals; n = 1 per stage). Samples were collected from Japanese elm (*Ulmus japonica*) in a valley elm–broadleaved forest, Ussuriysk Urban District, Primorsky Krai, Russia: larvae from decaying wood, adults from large-stemmed trees. The top 10 taxa are shown; remaining taxa are grouped as “Other”. Values are descriptive; no statistical comparison between stages was performed.

In the pooled samples, the taxonomic composition of the fungal component also differed between the adult and larval profiles (Figure 2). The total number of reads assigned to fungi was 5543 in the pooled larval sample and 504 in the pooled adult sample. This difference reflects library composition rather than absolute fungal load.

At the phylum level (Table S2d), Ascomycota was the dominant group in both stages; however, its proportion increased markedly in larvae, from 33.93% to 70.70%. Basidiomycota decreased from 31.75% to 21.23%, and Mucoromycota decreased from 23.41% to 4.17%. Microsporidia and Chytridiomycota were present in small amounts in both stages (6.94% and 1.32%; 3.97% and 2.58%, respectively).

At the class level (Table S3d), the most notable change was a sharp increase in the proportion of Saccharomycetes, from 8.33% in adults to 54.63% in larvae. Eurotiomycetes decreased from 20.04% to 9.74%, Pucciniomycetes from 14.88% to 3.05%, and Glomeromycetes from 16.87% to 3.01%, whereas Microbotryomycetes increased slightly from 9.13% to 11.15%. Tremellomycetes increased from 0% to 4.46%, and Sordariomycetes from 1.19% to 3.01%. The classes Chytridiomycetes, Dothideomycetes, Exobasidiomycetes, Mucoromycetes, Malasseziomycetes, and Agaricomycetes were present in small amounts (0.27–6.55%), whereas Leotiomycetes, Mixiomycetes, Saitoellomycetes, Schizosaccharomycetes, and Xylonomycetes were represented predominantly in larvae (0.20–1.30%).

At the order level (Table S4d), Saccharomycetales occupied the dominant position in larvae, increasing from 8.33% to 54.63%. Eurotiales decreased from 20.04% to 6.91%, Pucciniales from 14.88% to 3.05%, and Glomerales from 16.87% to 3.01%, whereas Sporidiobolales increased from 9.13% to 11.15%. Unclassified Microsporidia decreased from 6.94% to 1.32%. Tremellales (3.86%), Chaetothyriales (2.83%), and Ophiostomatales (2.40%) were present exclusively in larvae, whereas Pleosporales, Mortierellales, Exobasidiales, and Mucorales were substantially reduced. Malasseziales, Rhizophydiales, Polyporales, and Hypocreales were represented in both stages. The total proportion of other orders in larvae was about 8%.

At the family level (Table S5d), substantial changes were also observed: in adults, Aspergillaceae (20.04%), Glomeraceae (16.87%), Melampsoraceae (14.88%), and Sporidiobolaceae (9.13%) dominated, whereas in larvae, Saccharomycetaceae (from 2.98% to 31.48%) and Debaryomycetaceae (from 3.77% to 7.07%) increased sharply. Phaffomycetaceae (6.84%) and Pichiaceae (6.01%) were present exclusively in larvae, as were Cryptococcaceae (3.14%), Herpotrichiellaceae (2.83%), and Ophiostomataceae (2.40%). The proportion of other families increased from 7.14% in adults to 13.55% in larvae.

The most pronounced differences between the pooled profiles were observed at the genus level (Table S6d). In the pooled larval sample, *Kuraishia* had the highest relative abundance (29.57%) and was not detected in the pooled adult sample. *Rhodotorula* accounted for 11.15% in larvae versus 9.13% in adults. *Aspergillus* sharply decreased from 20.04% to 6.10%, *Rhizophagus* from 16.87% to 3.01%, and *Melampsora* from 14.88% to 2.62%. Genera such as *Ogataea* (4.93%), *Wickerhamomyces* (3.18%), *Komagataella* (3.05%), *Sporothrix* (1.86%), *Kwoniella* (1.84%), *Cryptococcus* (1.30%), *Babjeviella* (1.24%), *Ascoidea* (1.17%), *Candida* (1.12%), *Pichia* (1.08%), *Exophiala* (1.03%), and many others were present predominantly or exclusively in larvae. *Mitosporidium* decreased from 6.94% to 1.32%. The total proportion of other genera was 23.58% in the pooled larval sample compared with 7.14% in the pooled adult sample, consistent with the higher number of detected fungal genera in larvae.

### 3.4. Protist Component

**Figure 3:**
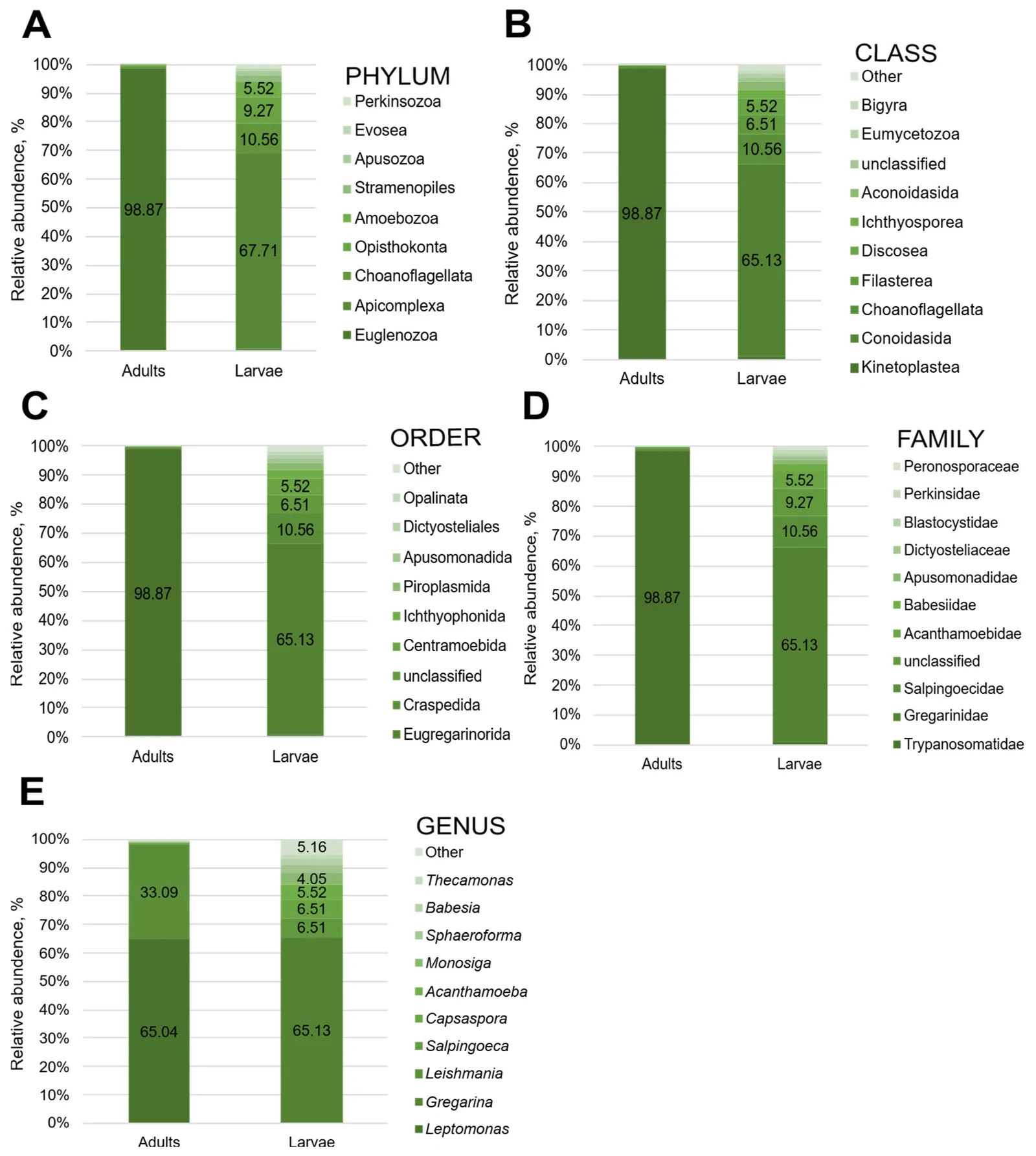
Taxonomic structure of the protist community in the gut of *Callipogon relictus* adults and larvae. Relative abundances (%) of protist taxa at the phylum (**A**), class (**B**), order (**C**), family (**D**), and genus (**E**) levels are shown for pooled samples (adults: two males and one female; larvae: three individuals; n = 1 per stage). Samples were collected from Japanese elm (*Ulmus japonica*) in a valley elm–broadleaved forest, Ussuriysk Urban District, Primorsky Krai, Russia: larvae from decaying wood, adults from large-stemmed trees. The top 10 taxa are shown; remaining taxa are grouped as “Other”. Values are descriptive; no statistical comparison between stages was performed.

In the pooled samples, the taxonomic composition of the protist component differed between the adult and larval profiles across all taxonomic levels (Figure 3). The total number of reads was 26,688 in the pooled adult sample and 1629 in the pooled larval sample. This difference reflects library composition rather than absolute protist load.

At the phylum level (Table S2c), Euglenozoa were the dominant group in adults (98.87%), whereas their proportion sharply decreased in larvae to 1.04%. In contrast, Apicomplexa were nearly absent in adults (0.09%) but became the absolute dominant in larvae (67.71%). Choanoflagellata increased from 0.45% to 10.56%, Opisthokonta from 0.38% to 9.27%, Amoebozoa from 0.05% to 5.52%, and Stramenopiles from 0.07% to 2.09%. Apusozoa, Evosea, and Perkinsozoa were present in small amounts (0.03–1.38%) in both stages.

At the class level (Table S3c), the dominance of Kinetoplastea in adults (98.87%) was replaced in larvae by a sharp decrease to 1.04%, whereas Conoidasida increased from 0.02% to 65.13%. Choanoflagellata, Filasterea, Discosea, and Ichthyosporea also increased substantially in larvae (from 0.05–0.45% to 2.76–10.56%). Unclassified protists from Apusozoa accounted for 1.35% in larvae, Eumycetozoa for 1.35%, Bigyra for 1.17%, Perkinsea for 1.10%, and Oomycota for 0.92%.

At the order level (Table S4c), Trypanosomatida, which dominated in adults (98.87%), decreased in larvae to 1.04%, yielding dominance to Eugregarinorida (from 0.02% to 65.13%). Craspedida increased from 0.45% to 10.56%, Centramoebida from 0.05% to 5.52%, Ichthyophonida from 0.10% to 2.76%, and Piroplasmida from 0.07% to 2.58%. Apusomonadida (1.35%), Dictyosteliales (1.35%), Opalinata (1.17%), Perkinsida (1.10%), and Peronosporales (0.92%) were present exclusively in larvae. Unclassified protists (*Capsaspora*) accounted for 6.51% in larvae.

At the family level (Table S5c), Trypanosomatidae decreased from 98.87% in adults to 1.04% in larvae, whereas Gregarinidae increased from 0.02% to 65.13%. Salpingoecidae increased from 0.45% to 10.56%, Acanthamoebidae from 0.05% to 5.52%, and Babesiidae from 0.07% to 2.58%. Apusomonadidae (1.35%), Dictyosteliaceae (1.35%), Blastocystidae (1.17%), Perkinsidae (1.10%), and Peronosporaceae (0.92%) were present exclusively in larvae. Unclassified families (*Capsaspora*, *Sphaeroforma*) accounted for 9.27% in larvae.

The most pronounced differences between the pooled profiles were observed at the genus level (Table S6c). In the pooled adult sample, the genera with the highest relative abundances were *Leptomonas* (65.04%) and *Leishmania* (33.09%), which were nearly absent in larvae (0.12% and 0.31%, respectively). *Trypanosoma* remained at a similar level (0.74% in adults, 0.61% in larvae). In the pooled larval sample, *Gregarina* showed the highest relative abundance (65.13%). *Salpingoeca* increased from 0.28% to 6.51%, *Capsaspora* from 0.28% to 6.51%, *Monosiga* from 0.16% to 4.05%, *Acanthamoeba* from 0.05% to 5.52%, *Sphaeroforma* from 0.10% to 2.76%, and *Babesia* from 0.07% to 2.58%. *Thecamonas* (1.35%), *Dictyostelium* (1.35%), *Blastocystis* (1.17%), *Perkinsus* (1.10%), and *Phytophthora* (0.92%) were present exclusively in larvae. The total proportion of other genera was extremely low in both stages.

### 3.5. Virus-like/Retroelement Sequences

The virus-like/retroelement fraction of the *C. relictus* microbiome exhibited pronounced differences between the pooled profiles (Figure S1). The total number of reads assigned to this fraction in adults was 7136, which was 4.0-fold higher than in larvae (1801 reads). In adults, the viral community was represented by a single phylum and genus, whereas larvae showed greater taxonomic diversity.

At all taxonomic levels (phylum to genus), this fraction was overwhelmingly dominated by a single lineage: Artverviricota (phylum)/Revtraviricetes (class)/Ortervirales (order)/Metaviridae (family)/*Errantivirus* (genus), accounting for 100.00% of the reads in adults and 97.45% in larvae (Tables S2d–S6d). The remaining 2.55% of the larval fraction comprised exclusively larval-specific taxa: the phyla Nucleocytoviricota and Uroviricota (classes Pokkesviricetes and Caudoviricetes; orders Chitovirales and Caudovirales), corresponding to the families Poxviridae and Autographiviridae, and the genera *Betaentomopoxvirus* and *Phikmvvirus* (1.28% each). These sequences may represent genuine viral or virus-like elements; however, their detection in total DNA does not distinguish free virions from integrated or endogenous elements. They are therefore reported here as virus-like sequences only.

### 3.6. Venn Diagram Analysis

**Figure 4:**
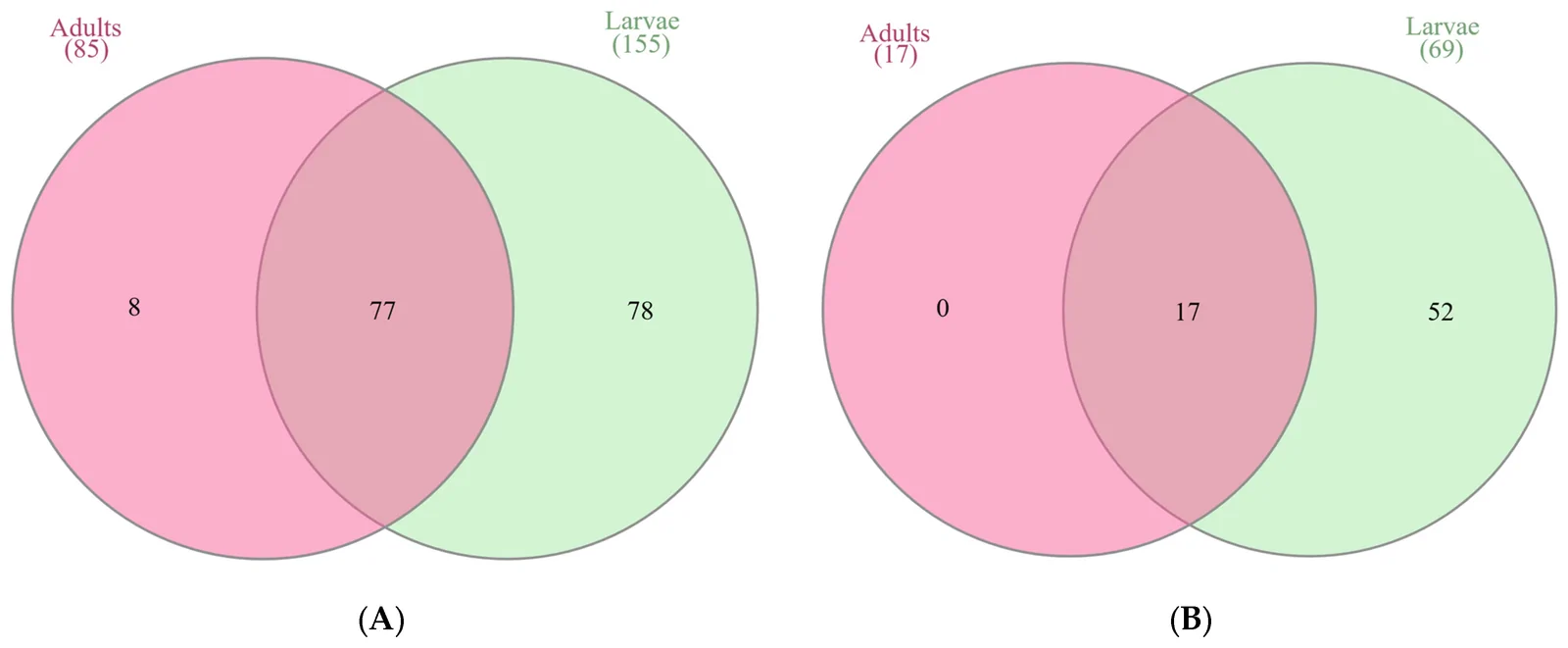
Venn diagrams showing the overlap of bacterial (**A**) and fungal (**B**) genera between adult and larval *Callipogon relictus* gut microbiomes. Numbers in circles indicate the numbers of unique and shared genera detected in pooled samples (adults: two males and one female; larvae: three individuals; n = 1 per stage). Samples were collected from Japanese elm (*Ulmus japonica*) in a valley elm–broadleaved forest, Ussuriysk Urban District, Primorsky Krai, Russia: larvae from decaying wood, adults from large-stemmed trees. The detection threshold was a relative abundance > 0.01%. Values are descriptive; no statistical comparison between stages was performed.

To assess the degree of similarity in the taxonomic composition of the microbiome between the adult and larval profiles of *C. relictus*, Venn diagrams were constructed at the level of bacterial and fungal genera (Figure 4). Venn diagrams for protists and virus-like/retroelement sequences are provided in Figure S2, with a complete summary of all taxonomic groups in Table S7. In the analysis of bacterial communities, duplicate entries for the same genus (arising from different strains or annotation variants) were merged to avoid artificial inflation of the number of taxa.

The Venn diagram for bacteria (Figure 4A) showed that 85 unique genera were detected in adult samples and 155 genera in larval samples. The intersection comprised 77 shared genera. Eight genera were unique to adults (e.g., *Morganella*, *Gibbsiella*, *Trabulsiella*, *Cedecea*, *Kosakonia*, *Providencia*, *Cosenzaea*, *Gilliamella*), whereas 78 genera were unique to larvae (including *Rickettsia*, *Methyloferula*, *Orientia*, *Robbsia*, *Nocardioides*, *Rhizobium*, and many others). Thus, approximately 50% of bacterial genera (78 out of 155) were unique to the pooled larval sample, consistent with the formation of a bacterial community adapted to the wood substrate in which larvae develop.

The analysis of fungal genera revealed even more pronounced differences between stages (Figure 4B). A total of 17 fungal genera were detected in adult samples and 69 genera in larval samples. The intersection comprised 17 shared genera; all genera identified in adults were also present in larvae. No genera were unique to adults (0), whereas 52 genera were unique to larvae (e.g., *Kuraishia*, *Ogataea*, *Wickerhamomyces*, *Komagataella*, *Cryptococcus*, *Candida*, *Pichia*, *Penicillium*, *Trichoderma*, and others). This represents more than 75% of all fungal genera detected in the pooled larval sample, consistent with the dominance of stage-specific fungal taxa in the larval profile, potentially associated with wood decomposition.

### 3.7. Alpha Diversity

To assess within-community diversity of the *C. relictus* microbiome, alpha diversity indices were calculated at the genus level for bacteria, fungi, protists, and virus-like sequences (Table 2). Calculations were based on pooled data for adults and larvae (one pool per stage) using the following metrics: richness (S; number of genera with non-zero read counts), Shannon index (H′, accounting for both richness and evenness of abundance distribution), Simpson index (1-D, reflecting the probability that two randomly selected reads belong to different genera), and Pielou’s evenness (J, indicating the degree of uniformity in the distribution of abundances among taxa).

In the pooled samples, bacterial alpha diversity indices were higher in the larval profile than in the adult profile: the number of detected genera was 85 in adults and 155 in larvae (1.8-fold), the Shannon index increased from 2.54 to 3.24, the Simpson index from 0.87 to 0.92, and evenness from 0.57 to 0.64. The fungal community showed the largest difference in alpha diversity between the pooled samples: the number of detected genera was 17 in adults and 69 in larvae (4.1-fold), the Shannon index from 2.18 to 3.11, and the Simpson index from 0.84 to 0.91, with similar evenness values (0.77 vs. 0.73), indicating that the increase in diversity was driven primarily by the appearance of new taxa.

The pooled samples had the same number of detected protist genera (15 each); however, the Shannon index was higher in the larval profile (1.58) than in the adult profile (0.60) (2.6-fold), the Simpson index increased from 0.37 to 0.70, and evenness from 0.22 to 0.58, reflecting a redistribution of abundances toward minor components while maintaining the same number of taxa. The virus-like/retroelement fraction showed very low alpha diversity: only one genus (*Errantivirus*) was detected in adults, and three genera in larvae; however, the dominance of *Errantivirus* (97.45%) persisted, resulting in a very low Shannon index (0.21).

### 3.8. Beta Diversity

To assess the degree of dissimilarity between the microbial communities of the adult and larval profiles of *C. relictus*, beta diversity indices were calculated based on genus-level data. Two presence/absence indices were used: the Jaccard index and the Sørensen index. In addition the Bray–Curtis dissimilarity index, was calculated for each taxonomic group. Calculations were performed separately for bacteria, fungi, protists, and virus like/retroelement sequences using data on the number of shared and unique genera and their absolute counts (Table 3). These indices are descriptive and based on pooled samples (n = 1 per stage); no statistical significance was assessed.

For bacteria, the Jaccard dissimilarity index was 0.528, indicating moderate dissimilarity in genus presence/absence: 77 genera were shared, whereas 8 genera were detected only in the pooled adult sample and 78 only in the pooled larval sample. The Bray–Curtis dissimilarity for bacteria was 0.872, indicating high dissimilarity in relative abundance between the pooled samples.

For fungi, the Jaccard index was 0.754, indicating high dissimilarity in presence/absence: all 17 genera detected in the pooled adult sample were also present in the pooled larval sample, and an additional 52 genera were detected only in the pooled larval sample. The Bray–Curtis dissimilarity for fungi was 0.834, also indicating high dissimilarity in relative abundance.

For protists, the Jaccard index was 0.000, indicating complete similarity in genus presence/absence: all 15 genera were shared between the pooled samples. However, the Bray–Curtis dissimilarity was 0.978, indicating very high dissimilarity in relative abundance, consistent with the strong shift from trypanosomatid-dominated sequences in the pooled adult profile to gregarine-dominated sequences in the pooled larval profile.

For virus-like/retroelement sequences, the Jaccard index was 0.667: only *Errantivirus* was shared between the pooled samples, whereas *Betaentomopoxvirus* and *Phikmvvirus* were detected exclusively in the pooled larval sample. The Bray–Curtis dissimilarity was 0.607, indicating moderate dissimilarity in relative abundance.

## 4. Discussion

The present data provide the first characterization of the bacterial, fungal, protist, and virus-like components of the gut microbiome of *C. relictus* based on shotgun metagenomic sequencing of pooled adult and larval samples. Bacterial sequences dominated in both larvae and adults, with Proteobacteria constituting the core phylum-level component; however, the relative proportions of classes and genera, as well as the structure of the fungal and protist fractions, differed markedly between the ontogenetic stages examined. The observed differences in the diversity and structure of gut microbial communities are consistent with data from other coleopteran insects and may be associated with metamorphosis-related changes in gut morphology, diet, and environmental contacts [23]. In adult beetles, the composition of bacterial and fungal communities is also linked to dietary specialization and habitat, and in Cerambycidae, to ontogenetic stage, host plant, and gut compartment [24,25]. Therefore, the differences identified here may result from the combined influence of developmental stage and the ecological conditions under which the *C. relictus* individuals were collected.

Analysis of the data showed that bacterial profiles were similar at the phylum level: Proteobacteria accounted for 87.88% of bacterial sequences in the pooled adult sample and 89.43% in the pooled larval sample. In the pooled adult sample, Gammaproteobacteria and Enterobacterales predominated, with the genera *Raoultella*, *Enterobacter*, *Klebsiella*, *Lactococcus*, *Pseudomonas*, and *Serratia* being the most abundant. In the pooled larval sample, the proportion of Alphaproteobacteria, particularly the orders Rickettsiales and Rhizobiales, was higher, and the share of Actinomycetota was 4.45% compared with 0.30% in the pooled adult sample; approximately 28% of bacterial sequences in the pooled larval sample were assigned to the genus *Rickettsia*.

Although the number of classified bacterial reads in the larval library was 3.7-fold lower, its taxonomic structure was characterized by a shift from the predominance of Gammaproteobacteria and Enterobacterales to Alphaproteobacteria and the orders Rickettsiales and Rhizobiales. The genera *Methyloferula* and *Orientia* were detected only in the pooled larval sample, whereas *Nocardioides*, *Rhizobium*, *Bradyrhizobium*, and *Neisseria* had higher relative abundances in the pooled larval sample. Genus-level richness in larvae was further increased by representatives of the phyla Acidobacteria, Verrucomicrobia, and Bacteroidetes. The predominance of Alphaproteobacteria, the increased proportion of Actinomycetota, and the higher number of genera are consistent with the influence of the wood substrate and the specific feeding ecology of larvae. Several larval-enriched taxa are potentially functionally relevant. In the pooled larval sample, *Methyloferula*, *Methylocella*, and *Methylovirgula* include methanotrophic species, which is consistent with the anaerobic, methane-producing environment of decaying wood [26]. *Rhizobium*, *Bradyrhizobium*, *Mesorhizobium*, and *Neorhizobium* include nitrogen-fixing species, consistent with a nitrogen-poor wood diet [27]. *Nocardioides* and other Actinomycetota include taxa known to produce cellulases, xylanases, and lignin-modifying enzymes, potentially contributing to lignocellulose degradation [28]. These inferences are consistent with metagenomic and metatranscriptomic studies of cerambycid larvae [29].

The high relative abundance of *Rickettsia* (27.96%) and the presence of *Orientia* (2.96%) in the pooled larval sample require cautious interpretation. Although *Rickettsia* are common intracellular symbionts in insects [30,31], their detection in whole-gut DNA does not distinguish between genuine colonization of the gut lumen and localization in host tissues. Because the analysis included the gut wall, sequences assigned to these genera may derive from tissue-associated or intracellular bacteria rather than from the luminal community. In addition, their ecological role—whether symbiotic, commensal, or pathogenic—cannot be inferred from taxonomic data alone. Therefore, the *Rickettsia*/*Orientia* signal should be considered preliminary and requires validation by independent methods such as qPCR or FISH to confirm both their presence and their localization.

The largest differences in taxonomic composition between the pooled profiles were observed in the fungal component. A total of 17 fungal genera were recorded in the pooled adult sample, compared with 69 in the pooled larval sample, and all genera detected in adults were also present in the larval profile. The number of classified fungal reads in the larval library was approximately 11-fold higher, and the predominance of the classes Eurotiomycetes and Pucciniomycetes in adults was replaced by the dominance of sac fungi of the class Saccharomycetes in larvae. Yeast representatives from the genera *Kuraishia*, *Ogataea*, *Wickerhamomyces*, *Komagataella*, *Sporothrix*, and *Kwoniella* were detected only in the pooled larval sample, consistent with a higher number of detected genera and a marked difference in taxonomic structure. The enrichment of saccharolytic yeasts in the larval gut is potentially functionally noteworthy. Species within Kuraishia, Ogataea, and Komagataella can ferment xylose and cellobiose derived from hemicellulose and cellulose, whereas Wickerhamomyces and Pichia include species possessing glycoside hydrolases and lipases [32,33,34]. These yeasts may therefore contribute to the breakdown of wood-derived carbohydrates and the detoxification of plant secondary metabolites, potentially complementing bacterial enzymatic activities [35]. In contrast, the pooled adult sample was dominated by *Aspergillus*, *Rhizophagus*, and *Melampsora*, which are more typical of environmental or plant-associated fungi than of the gut environment, suggesting that the larval fungal community is more closely linked to the wood substrate [32,33,36].

When comparing the two components, bacteria were numerically dominant in both pooled profiles (92.47% in adults, 85.31% in larvae), whereas fungi accounted for only 0.18% and 10.46%, respectively. However, the fungal component was taxonomically more diverse in relative terms (69 vs. 17 genera, a 4.1-fold difference) than the bacterial component (155 vs. 85 genera, a 1.8-fold difference). Functionally, bacteria are likely to be the primary drivers of nitrogen fixation and lignocellulose degradation, whereas fungi—particularly saccharolytic yeasts—may contribute mainly to hemicellulose breakdown and fermentation of wood-derived sugars [29]. The two groups may therefore act synergistically in the larval gut, with bacteria potentially providing nitrogen and fungi potentially providing carbohydrate-active enzymes [29]. This is consistent with the higher bacterial and fungal richness in the pooled larval sample, suggesting a potentially more functionally diverse community adapted to a wood-feeding lifestyle.

In *C. relictus*, the higher number of detected fungal genera in the pooled larval sample may reflect the prolonged contact of larvae with decaying elm wood; however, this interpretation requires further study. A similar pattern has been reported in other members of the family Cerambycidae [25,29]. The yeast component of the gut contents in longhorn beetles is highly diverse, but additional studies are required to distinguish resident forms from transient wood-associated taxa and to elucidate their transmission mechanisms [37].

The protist component was represented by sequences from 15 genera. In the pooled adult sample, trypanosomatid-related sequences assigned to the genera *Leptomonas* and *Leishmania* predominated, whereas in the pooled larval sample, gregarine-related sequences of the genus *Gregarina* were dominant. In addition, sequences from the classes Choanoflagellatea and Filasterea, the family Acanthamoebidae, and the genera *Sphaeroforma*, *Babesia*, *Thecamonas*, *Dictyostelium*, *Blastocystis*, *Perkinsus*, and *Phytophthora* were detected. The ecological roles of these protists remain largely unclear. Gregarines (*Gregarina*) are common extracellular parasites in the insect gut lumen, where they may affect host nutrition, immunity, and microbial competition. Their dominance in the pooled larval sample may reflect prolonged larval development in decaying wood, which may favour oocyst accumulation [38]. Trypanosomatids (*Leptomonas*, *Leishmania*), by contrast, are more typical of adult insects and may be acquired during feeding or flight [39,40,41].

In the virus-like fraction of both profiles, sequences of the genus *Errantivirus* strongly predominated; sequences assigned to the genera *Betaentomopoxvirus* and *Phikmvvirus* were detected only in the larval library. Importantly, the *Errantivirus* signal should not be interpreted as a gut virome. *Metaviridae* are LTR retrotransposons integrated into host genomes, and their detection in total DNA reflects endogenous retroelement sequences rather than free viral particles [42]. Distinguishing free viruses from endogenous elements would require virus-like particle (VLP) enrichment, RNA-based approaches, or RT-PCR validation, which were beyond the scope of this study.

Comparison with literature data on other longhorn beetles confirms a general pattern in the organization of the gut microbiome within the family Cerambycidae. Proteobacteria is the most frequently detected phylum, and Actinomycetota and Firmicutes also regularly occur in gut bacterial communities; among fungi, Ascomycota dominates in larvae. At the same time, the relative proportions of classes and genera vary with developmental stage, host plant, and microhabitat [24,29,43,44,45,46].

The pattern observed here—conservation of dominant high-rank taxa with pronounced variation at lower taxonomic ranks—is consistent with the general organization of cerambycid gut microbiomes reported in other species, where host plant, developmental stage, and microhabitat influence genus-level composition [24]. These results are consistent with the hypothesis of a two-level organization of the microbial communities in longhorn beetles: repeatability at the phylum and class levels across ontogenetic stages, and ecological variability (filtering) of the taxonomic composition at lower ranks.

The gut microbiome of *C. relictus* is likely shaped by developmental stage, wood substrate, and environmental microbial reservoirs. The higher bacterial and fungal richness in the pooled larval sample suggests a closer link to the wood substrate and a potentially more direct role in nutrient acquisition. This is relevant for conservation, as artificial diets in ex situ rearing may not provide the natural microbial community, potentially affecting larval development and fitness. Understanding microbiome acquisition in natural habitats is therefore important for the management of this rare species.

Several limitations must be acknowledged. First, each developmental stage was represented by a single pooled sample (n = 1 per stage), with no replicates. Consequently, no statistical comparisons were possible, and all reported differences are descriptive. The observed patterns may also reflect individual variation, sex composition, microhabitat, or other uncontrolled factors. Future studies with individual biological replicates are required to test the reproducibility of these patterns.

Second, pooling two males and one female into a single adult sample prevented assessment of sex-specific effects. Data from other beetles are inconsistent: sex influences the gut community in *Odontolabis fallaciosa* (Coleoptera: Lucanidae) [47], but not in *Carabus convexus* (Coleoptera: Carabidae) [48]. These differences appear to be taxon-specific, so dedicated studies using individually sequenced males and females are needed [49,50].

Third, larvae and adults were collected in different months (May and July) due to the species’ biology [12]. Seasonal factors—temperature, humidity, and changes in the substrate microbial community—cannot be excluded, and the relative contributions of developmental stage and season cannot be fully disentangled [51,52,53].

Fourth, the analysis was performed on the whole gut, including the gut wall, so the dataset may contain both luminal microbiota and tissue-associated or intracellular microbes [54,55].

Overall, this study is exploratory and descriptive, providing the first characterization of the *C. relictus* gut microbiome. The results generate hypotheses that require validation using replicated samples, individual sequencing, concurrent substrate and environmental sampling, and functional analyses.

## 5. Conclusions

This study provides the first shotgun metagenomic characterization of the gut microbiome of the endangered longhorn beetle *Callipogon relictus*, based on one pooled adult sample (two males and one female) and one pooled larval sample (three individuals). Both pooled profiles shared a bacterial core dominated by Proteobacteria but differed in lower-rank bacterial composition and in the structure of their fungal and protist components. The larval profile contained more detected bacterial and fungal genera, whereas the adult profile was characterized by higher relative abundances of Gammaproteobacteria and Enterobacterales. The protist profiles differed in dominance pattern, with trypanosomatid-related sequences predominant in adults and gregarine-related sequences predominant in larvae.

These findings provide a preliminary, descriptive basis for investigating developmental-stage-associated patterns in the gut microbiome of *C. relictus*. Because each stage was represented by a single pooled sample, the observed patterns cannot be statistically generalized and require validation using individually analyzed biological replicates, concurrent substrate sampling, and functional assays.

## Figures and Tables

**Table 1:** Overall structure of the gut microbiome of *Callipogon relictus* based on pooled samples (adults: two males and one female; larvae: three individuals).

Component	Adults	Larvae
Bacteria	92.47%	85.31%
Fungi	0.18%	10.46%
Protists	5.99%	2.53%
Virus-like/retroelement sequences	1.36%	1.70%
Archaea	0.00%	0.00%
Unidentified	<0.01%	<0.01%

**Table 2:** Alpha diversity of the *Callipogon relictus* microbiome by taxonomic group and developmental stage.

Component	Stage	S (Number of Genera)	Shannon Index (H′)	Simpson Index (1-D)	Evenness (J)
Bacteria	adults	85	2.54	0.87	0.57
	larvae	155	3.24	0.92	0.64
Fungi	adults	17	2.18	0.84	0.77
	larvae	69	3.11	0.91	0.73
Protists	adults	15	0.60	0.37	0.22
	larvae	15	1.58	0.70	0.58
Virus-like/retroelement sequences	adults	1	0.00	0.00	-
	larvae	3	0.21	0.11	0.19

Note: All samples were collected in a valley elm–broadleaved forest, Ussuriysk Urban District, Primorsky Krai, Russia, from Japanese elm (*Ulmus japonica*). The adult group consisted of two males and one female; the larval group consisted of three individuals. Values are point estimates based on pooled samples (n = 1 per stage). Values are descriptive; no statistical comparison between stages was performed.

**Table 3:** Beta diversity of the *Callipogon relictus* microbiome by taxonomic group.

Component	Shared Genera	Unique to Adults	Unique to Larvae	Jaccard Index (Dissimilarity)	Sørensen Index (Dissimilarity)	Bray–Curtis Dissimilarity
Bacteria	77	8	78	0.528	0.358	0.872
Fungi	17	0	52	0.754	0.605	0.834
Protists	15	0	0	0.000	0.000	0.978
Virus-like/retroelement sequences	1	0	2	0.667	0.500	0.607

Note: All samples were collected in a valley elm–broadleaved forest, Ussuriysk Urban District, Primorsky Krai, Russia, from Japanese elm (*Ulmus japonica*). The adult group consisted of two males and one female; the larval group consisted of three individuals. Values are point estimates based on pooled samples (n = 1 per stage). Values are descriptive; no statistical comparison between stages was performed.

## Data Availability

The raw data supporting the conclusions of this article will be made available by the authors on request.

## Supplementary Materials

The following supporting information can be downloaded at https://www.mdpi.com/article/10.3390/insects17101016/s1, Table S1: Sequencing and read mapping statistics for imago and larva samples of *Callipogon relictus*. Table S2a. Phylum-level taxonomic composition of bacterial communities in samples of *Callipogon relictus*. Table S2b. Phylum-level taxonomic composition of fungal communities in samples of *Callipogon relictus*. Table S2c. Phylum-level taxonomic composition of protozoan communities in samples of *Callipogon relictus*. Table S2d. Phylum-level taxonomic composition of virus-like/retroelement sequences in samples of *Callipogon relictus*. Table S3a. Clacc-level taxonomic composition of bacterial communities in samples of *Callipogon relictus*. Table S3b. Clacc-level taxonomic composition of fungal communities in samples of *Callipogon relictus*. Table S3c. Clacc-level taxonomic composition of protozoan communities in samples of *Callipogon relictus*. Table S3d. Clacc-level taxonomic composition of virus-like/retroelement sequences in samples of *Callipogon relictus*. Table S4a. Order-level taxonomic composition of bacterial communities in samples of *Callipogon relictus*. Table S4b. Order-level taxonomic composition of fungal communities in samples of *Callipogon relictus*. Table S4c. Order-level taxonomic composition of protozoan communities in samples of *Callipogon relictus*. Table S4d. Order-level taxonomic composition of virus-like/retroelement sequences in samples of *Callipogon relictus*. Table S5a. Family-level taxonomic composition of bacterial communities in samples of *Callipogon relictus*. Table S5b. Family-level taxonomic composition of fungal communities in samples of *Callipogon relictus*. Table S5c. Family-level taxonomic composition of fungal communities in samples of *Callipogon relictus*. Table S5d. Family-level taxonomic composition of virus-like/retroelement sequences in samples of *Callipogon relictus*. Table S6a. Genus-level taxonomic composition of bacterial communities in samples of *Callipogon relictus*. Table S6b. Genus-level taxonomic composition of fungal communities in samples of *Callipogon relictus*. Table S6c. Genus-level taxonomic composition of protozoan communities in samples of *Callipogon relictus*. Table S6d. Genus-level taxonomic composition of virus-like/retroelement sequences in samples of *Callipogon relictus*. Table S7: Summary of Venn diagram analysis showing the distribution of genera across taxonomic groups in adult and larval *Callipogon relictus* microbiomes. Figure S1: Taxonomic structure of the virus-like/retroelement sequence fraction in the gut of *Callipogon relictus* adults and larvae. Relative abundances (%) of taxa at the phylum (A), class (B), order (C), family (D), and genus (E) levels are shown for pooled samples (adults: two males and one female; larvae: three individuals; n = 1 per stage). Samples were collected from Japanese elm (*Ulmus japonica*) in a valley elm–broadleaved forest, Ussuriysk Urban District, Primorsky Krai, Russia: larvae from decaying wood, adults from large-stemmed trees. The top taxa are shown; remaining taxa are grouped as “Other”. Values are descriptive; no statistical comparison between stages was performed. Figure S2: Venn diagrams showing the overlap of genera between adult and larval *Callipogon relictus* gut microbiomes for protists (A) and virus-like/retroelement sequences (B). Numbers in circles indicate the numbers of unique and shared genera detected in pooled samples (adults: two males and one female; larvae: three individuals; n = 1 per stage). The detection threshold was a relative abundance > 0.01%. Values are descriptive; no statistical comparison between stages was performed.
